# Access to information and oncofertility consultation for young women with breast cancer: a population-based study

**DOI:** 10.1038/s41598-023-30423-3

**Published:** 2023-02-27

**Authors:** Florian Martinet-Kosinski, Sébastien Lamy, Eric Bauvin, Florence Dalenc, Charlotte Vaysse, Pascale Grosclaude

**Affiliations:** 1 CERPOP UMR 1295, Paul Sabatier University Toulouse III, Inserm, Team Labelled By the French League Against Cancer, Toulouse, France; 2 Group for Research and Analysis in Population Health (GAP), Claudius Regaud Institute, Toulouse University Cancer Institute (IUCT-O), Toulouse, France; 3 Tarn Cancers Registry, Claudius Regaud Institute, Toulouse University Cancer Institute (IUCT-O), Toulouse, France; 4 Occitanie Regional Cancer Network (Onco-Occitanie), Toulouse, France; 5 Department of Oncology, Claudius Regaud Institute, Toulouse University Cancer Institute (IUCT-O), Toulouse, France; 6 Department of Gynecologic Surgery, Toulouse University Hospital, Toulouse University Cancer Institute (IUCT-O), Paul Sabatier University Toulouse III, Toulouse, France

**Keywords:** Cancer epidemiology, Breast cancer, Endocrine reproductive disorders, Risk factors

## Abstract

Non-menopausal women with breast cancer treated with chemotherapy are at intermediate risk of post-treatment amenorrhea and decreased fertility. Although they should receive appropriate information, studies until now show that this is inadequate. We investigated the proportion of women who received information about this risk during the pre-treatment consultation, and those who received an oncofertility consultation to preserve their gametes. We also analysed the medical and non-medical factors influencing the transmission of information to patients and their uptake of oncofertility consultations. We included women aged 18–40 years treated with chemotherapy for breast cancer between 2012 and 2017 in the Midi-Pyrénées region (ca. 3 million inhabitants), France. Studied variables were included in a multilevel model. Among the 575 women, 41% of the women received information and 28% received an oncofertility consultation. These two steps on the care pathway were significantly influenced by the type of care structure, the woman's age, her parity at the time of diagnosis, and the metastatic status of the cancer. Female oncologist gender was significantly associated with higher transmission rate. We found no association between neoadjuvant chemotherapy status, level of deprivation (EDI), triple-negative status, marital status, and first-degree family history of cancer and information transmission or uptake of oncofertility consultation. Our study shows that not enough women are informed and have recourse to an oncofertility consultation. Despite a legal obligation, the health care system does not offer the necessary conditions for access to oncofertility care.

## Introduction

As cancer therapies are becoming more effective and curative, cancer-related issues are no longer solely focused on survival^1^ but now take into account the quality of life after treatment^2^. Therefore the question of oncofertility is becoming increasingly important in cancer management. In current clinical practice in France, during a consultation prior to cancer treatment, the oncologist must inform the patient about the gonadotoxicity of chemotherapies and must propose a consultation in a specialized centre to discuss fertility preservation feasibility. During this consultation, the gynaecologist assesses the patient's ovarian reserve, explains the impact of the treatments on fertility and presents the different possible preservation techniques^3^. Then the gynaecologist registers the patient for a multidisciplinary team meeting where the physicians validate the indication for preservation and then the technique used^4^.

In France, since 2004, there has been a legal obligation to propose fertility preservation to patients before any anti-tumoural treatment associated with gonadotoxicity^5^. Fertility preservation must be considered as an important option for young cancer patients^6^ and oncologists^7^, but still seems to be insufficiently discussed in consultations with oncologists. Very few studies have been conducted to find out how this information, and the resulting proposal for gamete preservation, is given. Furthermore, a preliminary study carried out in the region showed a great disparity in access to fertility oncology consultation^8^.

We hypothesize that improving access to fertility preservation services depends largely on access to information about the risk of gonadotoxicity associated with certain cancer treatments, and the existence of possible solutions for fertility preservation and their specificities.

## Objectives

The primary objective was to establish what proportion of women received information about the risk of chemotherapy-related fertility impairment during the disclosure consultation, and what proportion had an oncofertility consultation. The secondary objective was to analyse the medical and non-medical factors associated with having been informed and having received the specialist oncofertility consultation.

## Materials and methods

### Study population

**Figure 1:**
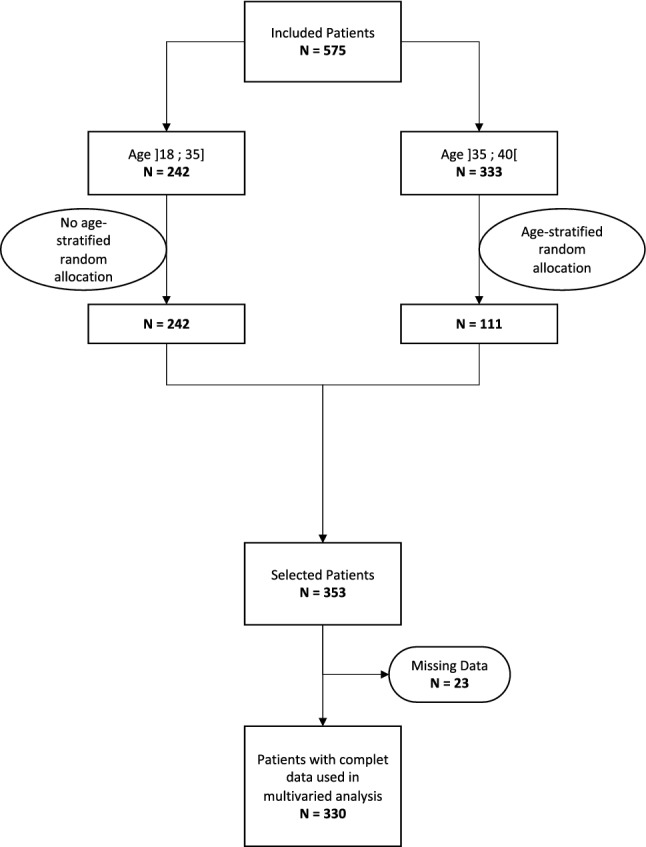
Flowchart.

This study concerned women aged 18–40 years with invasive breast cancer eligible for (neo)adjuvant chemotherapy, diagnosed between January 2012 and December 2017 in the Midi-Pyrénées region, France. We identified the patients through the regional cancer network's communicating cancer file, which centralizes all multidisciplinary team meeting (MTM) files in the region. The effective administration of chemotherapy was verified in the MTM files by checking the lists of treatments delivered by the hospital pharmacies, or by contacting the oncology departments directly. Women for whom this verification process could not be performed were excluded. In order to have a sufficient representation of young women while maintaining a reasonable survey sample size all women aged ≤ 35 years (n = 242) were included in the study and one in three women between the ages of 36 and 40 years were selected at random (n = 111), resulting in a sample of 353 patients (Table 1 and Fig. 1). Given the missing data on the main variables, the number of cases included in the study was 330 (see Fig. 1 and Appendix 1A for their description). We did not contact the women enrolled in the study afterwards to verify the data found in the medical records.

**Table 1:** Association of variables with information provision and oncofertility consultation (bivariate analysis).

Variables	All cases		Fertility information					Oncofertility consultation				
			Yes		No		p	Yes		No		p
	N*	%	N*	%	N*	%		N*	%	N*	%	
Total number of patient												
	536	1.00	220	0.41	316	0.59		151	0.28	385	0.72	
Age												
19–24 years	10	0.02	6	0.60	4	0.40	< 0.001	5	0.50	5	0.50	< 0.001
25–29 years	47	0.09	36	0.77	11	0.23		34	0.72	13	0.28	
30–34 years	162	0.30	100	0.62	62	0.38		71	0.44	91	0.56	
35–39 years	239	0.45	66	0.28	173	0.72		38	0.16	201	0.84	
40 years	78	0.15	12	0.15	66	0.85		3	0.04	75	0.96	
MD	0											
Hospital type												
Teaching Hospital	338	0.63	176	0.52	162	0.48	< 0.001	118	0.35	220	0.65	< 0.001
Private Toulouse	124	0.23	33	0.27	91	0.73		27	0.22	97	0.78	
Private outlying	44	0.08	3	0.07	41	0.93		3	0.07	41	0.93	
Public outlying	30	0.06	8	0.27	22	0.73		3	0.10	27	0.90	
MD	0											
Oncologist gender												
Female	306	0.57	154	0.50	152	0.50	< 0.001	103	0.34	203	0.66	0.015
Male	230	0.43	66	0.29	164	0.71		48	0.21	182	0.79	
MD	0											
Number of children												
0	123	0.23	89	0.72	34	0.28	< 0.001	82	0.67	41	0.33	< 0.001
1	120	0.22	58	0.48	62	0.52		49	0.41	71	0.59	
2	210	0.39	59	0.28	151	0.72		16	0.08	194	0.92	
3+	83	0.15	14	0.17	69	0.83		4	0.05	79	0.95	
MD	0											
Metastatic												
No	493	0.92	213	0.43	280	0.57	0.01	48	0.13	336	0.88	< 0.001
Yes	43	0.08	7	0.16	36	0.84		2	0.05	41	0.95	
MD	0											
EDI												
Quintile 1	132	0.25	53	0.40	79	0.60	0.75	37	0.28	95	0.72	0.78
Quintile 2	119	0.22	41	0.34	78	0.66		28	0.24	91	0.76	
Quintile 3	95	0.18	41	0.43	54	0.57		28	0.29	67	0.71	
Quintile 4	103	0.19	48	0.47	55	0.53		28	0.27	75	0.73	
Quintile 5	84	0.16	36	0.43	48	0.57		29	0.35	55	0.65	
MD	3											
CT neoadjuvant or 1st line												
No	317	0.60	125	0.39	192	0.61	0.40	89	0.28	228	0.72	0.84
Yes	213	0.40	95	0.45	118	0.55		62	0.29	151	0.71	
MD	6											
Year of diagnosis												
2012	108	0.20	27	0.25	81	0.75	0.14	13	0.12	95	0.88	0.04
2013	88	0.16	43	0.49	45	0.51		33	0.38	55	0.63	
2014	85	0.16	39	0.46	46	0.54		24	0.28	61	0.72	
2015	93	0.17	38	0.41	55	0.59		30	0.32	63	0.68	
2016	105	0.20	44	0.42	61	0.58		32	0.30	73	0.70	
2017	57	0.11	29	0.51	28	0.49		19	0.33	38	0.67	
MD	0											
Triple-negative												
Yes	158	0.29	55	0.35	103	0.65	0.16	111	0.29	267	0.71	0.46
No	378	0.71	165	0.44	213	0.56		40	0.25	118	0.75	
MD	0											
Marital status												
Married	379	0.77	161	0.42	225	0.58	0.48	103	0.27	283	0.73	0.10
Alone	116	0.23	55	0.47	62	0.53		44	0.38	73	0.62	
MD	33											
Family history												
Yes	109	0.21	170	0.73	62	0.27	0.93	30	0.28	79	0.72	0.75
No	400	0.79	47	0.17	230	0.83		118	0.30	282	0.71	
MD	27											

N* weighted number of cases, *P* P-value, *MD* missing data, *CT* chemotherapy.

### Variables

The same person collected the data in the various private and public hospitals.

#### Main outcomes

The outcomes were whether the women had been informed of the risk of decreased fertility and whether they had an oncofertility consultation. Outcomes were classified as binary variables. We considered a woman to have been informed if it was mentioned in the report of the disclosure consultation. For the specialized consultation, we assumed that a woman had consulted if she was listed in the two oncofertility centres in the Midi-Pyrénées region.

#### Main explanatory variables

We studied factors related to the care pathway, including the care facility and the oncologist who managed the patient. The care hospitals were classified into four groups according to their location or not in the regional capital and whether they were public or private: teaching hospital, Toulouse private hospital, outlying public hospital; outlying private hospital. The only oncologist-related characteristic was woman or man. Patient characteristics were as follows: age in years, number of children (1, 2 and 3 or more), marital status at diagnosis, family history of breast cancer, and social conditions as assessed by the European Deprivation Index (EDI)^9^. Cancer-related characteristics were as follows: year of diagnosis, metastatic tumour or not, triple-negative tumour or not, and neoadjuvant or 1st line chemotherapy vs not.

### Statistical analysis

To address our primary objective, we first assessed the proportion of women who had been informed about the chemotherapy-related infertility risk, and second, the proportion of those who had received a fertility consultation. These proportions were obtained in a weighted sample to account for sample rate associated with women older than 35 years, who were three-fold less numerous than those in the general population due to our inclusion strategy. The population size was 330 women and increased to 575 women when the sample design was taken into account. To address our second objective, we modelled the probability of each outcome, i.e. having been informed of the chemotherapy-related infertility risk and having received a fertility consultation, in relation to the factors associated with it. First, the factors potentially associated with each outcome were tested in bivariate analyses. Then, we built multivariable models to assess the independent association between the factors retained from the bivariate analyses and each outcome. Regarding the binary nature of the outcomes and the hierarchical structure of the data, wherever different women may have had the same oncologist, we used a mixed-effect generalized linear model with logit link function and a random intercept to model the inter-oncologist variability in the outcome probability. In both bivariate and multivariable analyses, models were adjusted for age. In the multivariable model, the variables were adjusted for sequentially, considering patient-associated characteristics before oncologist-associated variables, as recommended in multilevel models^10^. We defined the analysed sample as the women who had complete data on the variables that were adjusted for in the multivariable models. Results of both bivariate and multivariable analyses are shown for the analysed sample. All analyses except those in multivariable models were done on the weighted sample. Multilevel multivariable (including age) analyses were performed on the unweighted sample to prevent computational issues. We reported patients’ and oncologists’ characteristics for both the analysed sample and the women who were excluded from the analyses. Statistical significance was set at 0.2 and 0.05 for bivariate and multivariable analyses, respectively. All analyses were done using STATA software (StataCorp LP, College Station, TX, version 11.1).

### Ethics approval

The processing of information collected is the subject of a declaration to CNIL, the French data protection agency, under no. 917235.

### Consent to participate

All participants were informed about the use of their data in this study and had the possibility to refuse in accordance with the law.

### Legal statement

In accordance with the directives of the French regulation. The protocol has been approved by CEREES N° INDS : TPS 391315bis. The protocol has been approved by the CNIL: Notification N° 917235v1. In accordance with this CNIL notification, patients were informed individually of the study and of their right to object to the use of their data.

## Results

### Description of population (Table 1)

On the weighted sample, most of the women had been treated in the teaching hospital (63%). Women treated in an outlying hospital represented only 14% of the population. More than half (57%) of the women in the study were managed by a female oncologist. Regarding the characteristics of the women, 41% of the women in the study were under 35 years of age, 23% were nulliparous, 27% were living with a partner at the time of diagnosis.

Regarding cancer-related data, 92% of the women had no metastases at the time of diagnosis, 60% did not receive neoadjuvant chemotherapy, 29% had triple-negative status.

The weighted analysis showed that the oncologists noted having informed 41% (n = 220) of the women about the risks of decreased fertility after chemotherapy. Only 28% of the women had had an oncofertility consultation (n = 151). Of the 220 women who received the information, 68% went to the oncofertility consultation.

Age at diagnosis was associated with discussing the risk of impaired fertility and with having an oncofertility consultation. Older women were less likely to have been informed and to have received oncofertility counselling. The same tendency was noted for parity: the more children that women had, the less their oncologists had discussed the risk of fertility impairment due to chemotherapy and the less they had had the opportunity of oncofertility counselling. Having metastatic cancer at the time of the disclosure reduced the transmission of information and the uptake of oncofertility consultations. Regarding the year of diagnosis, there was a clear increase in the transmission of information and uptake of consultations from 2013. On the other hand, neither adjuvant versus neoadjuvant or 1st line chemotherapy, level of deprivation, marital status, having a first-degree family history of breast cancer, or triple-negative status of the tumour were associated with the transmission of information during the cancer disclosure consultation nor with having the oncofertility consultation. The type of care facility the patient attended was associated with receiving fertility information and having the oncofertility consultation since women treated in the Toulouse teaching hospital were significantly more informed and had more recourse to the oncofertility consultation. Moreover, when the oncologist was a woman, the patient was more often informed about the risk of infertility than when he was a man. However, this difference was less marked regarding the access to of oncofertility counselling.

### Multilevel analysis (Tables 2, 3)

**Table 2:** Factors associated with transmission of fertility information (multilevel analysis).

Variables	N		N	AIC	Loglik	N	AIC	Loglik	N	AIC	Loglik
	330		330		− 212.18	330		− 163.97	330		− 156.58
Oncologist			0.21		[0.07; 0.45]	0.26		[0.10; 0.52]	0.15		[0.04; 0.40]
Patient characteristics											
Patient age ©	0.83	0.000					0.87	[0.80; 0.94]		0.87	[0.81; 0.94]
Number of children ©	0.57	0.000					0.42	[0.29; 0.59]		0.42	[0.30; 0.59]
Non-metastatic	1						1			1	
Metastatic	0.14	0.000					0.06	[0.02; 0.23]		0.06	[0.02; 0.23]
Year of diagnosis ©	1.20	0.01					1.39	[1.15; 1.68]		1.36	[1.12; 1.64]
Hospital type											
Teaching Hospital	1									1	
Private Toulouse	0.34	0.001								0.44	[0.16; 1.23]
Private outlying	0.09	0.001								0.11	[0.02; 0.55]
Public outlying	0.27	0.020								0.33	[0.08; 1.32]
Oncologist gender											
Female oncologist	1	0.000								1	
Male oncologist	0.42									0.39	[0.15; 1.01]

*N* unweighted number of cases, *©* continuous variable, *OR* Odds ratio, *loglik* log-likelihood ratio, *AIC* Akaike information criterion, *ICC* intraclass correlation coefficient, *95% IC* 95% confidence interval.

**Table 3:** Factors associated with having had an oncofertility consultation (multilevel analysis).

Variables	N		N	AIC	Loglik	N	AIC	Loglik	N	AIC	Loglik
	330		330		− 212.23	330		− 144.11	330		− 139.29
	OR	p-value	ICC	OR	95% IC	ICC	OR	95% IC	ICC	OR	95% IC
Oncologist			0.25		[0.09 ; 0.53]	0.33		[0.11; 0.65]	0.27		[0.08; 0.60]
Patient characteristics											
Patient age ©	0.81	0.000					0.86	[0.79; 0.94]		0.86	[0.79; 0.94]
Number of children ©	0.36	0.000					0.25	[0.16; 0.37]		0.24	[0.16; 0.37]
Non-metastatic	1						1			1	
Metastatic	0.076	0.001					0.02	[0.003; 0.14]		0.02	[0.004; 0.14]
Year of diagnosis ©	1.17	0.032					1.31	[1.07; 1.62]		1.28	[1.04; 1.58]
Hospital type											
Teaching Hospital	1									1	
Private Toulouse	0.57	0.075								0.87	[0.23; 3.13]
Private outlying	0.21	0.021								0.18	[0.26; 1.26]
Public outlying	0.20	0.022								0.15	[0.02; 0.99]
Oncologist gender											
Female oncologist	1									1	
Male oncologist	0.69	0.145								0.39	[0.11; 1.34]

*N* unweighted number of cases, *©* continuous variable, *OR* Odds ratio fixed effect, *log lik* log-likelihood ratio, *AIC* Akaike information criterion, *ICC* intraclass correlation coefficient, *95% IC* 95% confidence interval.

Multilevel analysis was carried out on the unweighted sample (330 women) (Table 2). The probability of receiving information varied according to the oncologist (ICC (Interclass Correlation Coefficient) = 0.21 in the empty model) and increased when the characteristics of the woman (age, number of children) or her tumour (metastasis, year of diagnosis) were taken into account. All these characteristics were strongly associated with the fact of having been informed or not, (less information for the oldest women or those with more children, as well as for women with metastatic cancer). These inter-physician differences cannot thus be explained by the characteristics of the women at the beginning of their care trajectory (ICC = 0.26). On the other hand, the type of care facility attended and the oncologist’s gender did have an effect. The inter-oncologist variability was partially explained by the type of hospital attended and the oncologists’ gender (ICC = 0.15 in the full model). Being treated in a public hospital in Toulouse rather than elsewhere increased the chances of receiving information about fertility problems.

The crude probability of having access to an oncofertility consultation (Table 3) also varied between oncologists (ICC = 0.25). It increased strongly when accounting for the characteristics of the patients and their tumour, reflecting differences between the oncologists’ patients (ICC = 0.33). The inter-oncologist variability was partially explained by the type of hospital attended (ICC = 0.27 when the care setting and the oncologist’s gender were taken into account in the full model). Women treated in the teaching hospital in Toulouse were more likely to have access to an oncofertility consultation, especially when compared to those treated outside Toulouse.

## Discussion

Our study is one of the few to provide a global view of the transmission of information about the risks of decreased fertility and the access to oncofertility consultations on a regional scale. Only 41% of women aged 40 years or under were informed about the risk of infertility and the proportion of women receiving an oncofertility consultation was 28%. There was an increase in information transmission and consultation uptake between 2012 and 2017. This increase can be explained by the fact that the second French national cancer plan, which was launched in 2009, reiterated the fact that providing information about the risk of post-treatment infertility is a major issue in patients recovering from cancer treatment.

Studies documenting access to fertility counselling and referral to fertility specialists have produced very different results depending on the period considered, the survey response rate, the geographical context, the cultural context, the study design, and the outcome of interest. In a US survey of cancer survivor diagnoses between 1999 and 2009 in the State of Georgia, 59% of responders reported having been counselled on the infertility risk associated with both their cancer and its treatment, but the response rate in that survey was only 25%^11^. The rate of fertility information exceeded 80% in a more recent US study of the 2010–2012 medical records of a comprehensive centre in which a full-time patient navigator was dedicated to fertility preservation information and coordination^12^, and 62% in a single-centre US study on the 2009–2013 medical records of a large private academic medical centre^13^. In Europe, a Dutch retrospective study showed that although the absolute number of patients receiving fertility preservation counselling increased over time, only 9.8% of all potential patients aged under 40 and managed in a teaching hospital in 2011 were referred for counselling^14^. An Ontario registry study of young women aged 15–39 years with breast cancer diagnosed between 2000 and 2016 found an average infertility consultation rate of 4% over the entire period. However, the rate increased steadily over time starting from less than 1% in 2000 to over 10% after 2014^15^. Another Ontario registry study of lymphoma cases diagnosed between 2000 and 2018 in young patients aged 15–39 years found a steadily increasing infertility consultation rate from 1% between 2000 and 2006 to 7.9% between 2014 and 2018^16^. More recently, the PREFER study, an observational, prospective study enrolling premenopausal women with early breast cancer, shows that after being informed of the risks associated with chemotherapy a complete reproductive counseling conducted at the fertility unit was accepted by 34,6% of women aged between 18 and 40 years^17^. The VICAN study, about French cancer survivors 2 and 5 years after cancer diagnosis in 2010, 32.6% of women reported that FP counseling had been provided to them before cancer treatment^18^.

Like ours, all these studies found that the higher the age and parity of the woman at the time of diagnosis, the lower the use of information and uptake of consultations. This could be because much information must be given during the disclosure consultation. In an older woman and/or one who has already had children, the risk of infertility seems to be less of a priority.

Our study has some limitations. First, it may have slightly underestimated the amount of information given to patients. It was sometimes difficult to trace the transmission of information by the oncologist. Since this information was in the patients’ records, we assumed that the physician had discussed possible fertility problems related to chemotherapy with them. However, the giving of information may not have been noted down at any moment, either because the physician forgot to note it in the consultation report or because it was included in another document to which we did not have access. The fact that the frequency of missing data was significant for other variables that are associated with a low rate of patient information is consistent with this (Appendix 1A). Data were more frequently missing in private hospitals, and information about patients seemed to have been transmitted less often in these institutions. However, the importance of this bias should be put into perspective because there was also an association between the proportion of missing data and not having received an oncofertility consultation. Nevertheless, unlike information given to a patient, the existence of a consultation could be objectively established since we cross-referenced our files with those of the only two fertility centres in the region.

Another limitation is that we did not consider the women's desire to have a child, which is a fundamental consideration before deciding on gamete preservation. Unfortunately, there was very little information about this in the medical records and we were unable to use it for our analysis. Furthermore, we did not wish to question the women directly for reasons. We think it would have been difficult to discuss the risk of decreased fertility a posteriori with women who had not previously received any information about it. The fact that we did not question the women directly did not allow us to check whether they had been correctly informed, nor their desire for a child at the time of diagnosis.

Regarding social deprivation, we did not find a significant link between the level of deprivation and information transmission or consultation uptake. On the other hand, Mahey et al.^19^ found that women's risk-related knowledge was low and varied along the socioeconomic gradient. Furthermore, in a retrospective study of 2012, Letourneau et al. showed that women without a bachelor’s degree were less aware of infertility risks^20^. The lack of significance on this factor in our study may be because we used an ecological index of deprivation and not an individual indicator. However, we did find geographical inequalities, since women treated in hospitals located in the regional capital were much more informed and had a much higher rate of consultation uptake than those treated in outlying centres. This is probably because the only two centres in the region that perform gamete preservation are located in Toulouse.

We hypothesised that a short delay between diagnosis and the initiation of chemotherapy, which is the case if chemotherapy is the first treatment, and particularly in the event of neoadjuvant chemotherapy, might be a barrier to the implementation of fertility preservation. Our results do not support this hypothesis as we did not observe any association between receiving neoadjuvant chemotherapy and information transmission or consultation uptake. A recent meta-analysis demonstrate that performing fertility preservation after diagnosis do not seem to worsen the prognosis of breast cancer in young patients but, as the author of the study point out, there is a risk of bias in the selection of patients with favorable prognostic characteristics^21^. We also examined the association with triple-negative status, which was also not associated with information transmission or consultation uptake. In contrast, women with early metastatic cancer were significantly less informed and made less use of counselling. These women may have considered that discussing fertility preservation would have been a waste of "precious" time when faced with a poor prognosis. However, French law stipulates that all women must be informed. Thus, this obligation to inform may seem questionable in this case^22^. On the other hand, more teams agree that this should also be offered to women who are going to receive hormonotherapy alone. This will delay the project of becoming pregnant by 3–5 years, making it more difficult for these women to have a child owing to their advancing age and reduced fertility^23^.

The amount of information transmitted, and therefore the uptake of consultations, depends on oncologists. This oncologist effect is partly related to the profile of the patients they treat, but it is also associated with the type of hospital in which they work. Women who were treated in the teaching hospital, and more generally those who were treated in the regional capital, were more likely to be informed and to have a consultation than the others. The oncologist effect also seemed to depend on whether the doctor was a man or a woman, especially with regard to giving information about the risk of infertility. These results are in agreement with Shimuzi's study, which showed that young oncologists and female oncologists were more likely to refer their patients to a reproductive specialist^24^. This suggests that strategies are needed to mitigate these deficits in the access to fertility preservation. Several decision-aid tools exist for patients, e.g. in Australia^25^, Canada^26^, UK^27^, and Europe^28,29^. In Canada, a quasi-experimental study compared the rate of patients reported as having been informed of fertility issues between two academic centres, only one of which had a nurse navigator-based program dedicated to young cancer patients. Both a higher self-reported information rate (+ 20%) and a high rate of referral to fertility preservation (+ 40%) were found in the centre with the nurse navigator program than in the centre where no such intervention existed. The nurse navigator screened referrals to the cancer centre, contacted all women aged 40 years or less prior to or at their initial appointment and followed them during diagnosis, treatment and beyond, especially in raising age-related issues including fertility, genetics and sexual health^30^. More recently, a multicomponent randomized trial compared consultation and referral if requested to the combination of provider education, patient decision aid, and navigation support^27,31^.

## Conclusion

Our findings show that when the treatment is announced after their diagnosis, many women are not informed about the risk of gonadotoxicity associated with certain anticancer treatments, nor about the pros and the cons of the possible solutions for preserving their fertility. Consistent with Andersen’s concept of facilitator^32^ and McCullock Melnyk’s notion of barrier to access to care^33^, we hypothesize that improving access to gamete preservation services depends largely on access to this information.

For this reason, our regional cancer network has published a set of brochures for practitioners and patients. A computerised alert is also given to young women after the MTM. The impact of these new tools now needs to be evaluated, both on the information provided and on the reduction of territorial inequalities that the present study has revealed.

## Supplementary Information

Supplementary Information 1.

Supplementary Information 2.

Supplementary Information 3.

## Data Availability

The datasets generated during and/or analysed during the current study are available from the corresponding author on reasonable request.
